# Decoding cancer prognosis with deep learning: the ASD-cancer framework for tumor microenvironment analysis

**DOI:** 10.1128/msystems.01455-24

**Published:** 2025-04-16

**Authors:** Ziyuan Huang, Yunzhan Li, Vanni Bucci, John P. Haran

**Affiliations:** 1 Department of Emergency Medicine, UMass Chan Medical School, Worcester, Massachusetts, USA; 2 Department of Microbiology, UMass Chan Medical School, Worcester, Massachusetts, USA; 3 Department of Cellular and Molecular Physiology, Penn State College of Medicine, Hershey, Pennsylvania, USA; 4 Program in Microbiome Dynamics, UMass Chan Medical School, Worcester, Massachusetts, USA; Georgia Institute of Technology, Atlanta, Georgia, USA

**Keywords:** deep learning, autoencoder, transfer learning, multi-omics, cancer prognosis

## Abstract

Deep learning is revolutionizing biomedical research by facilitating the integration of multi-omics data sets while bridging classical bioinformatics with existing knowledge. Building on this powerful potential, Zhang et al. proposed a semi-supervised learning framework called Autoencoder-Based Subtypes Detector for Cancer (ASD-cancer) to improve the multi-omics data analysis (H. Zhang, X. Xiong, M. Cheng, et al., 2024, mSystems 9:e01395-24, https://doi.org/10.1128/msystems.01395-24). By utilizing autoencoders pre-trained on The Cancer Genome Atlas data, the ASD-cancer framework outperforms the baseline model. This approach also makes the framework scalable, enabling it to process new data sets through transfer learning without retraining. This commentary explores the methodological innovations and scalability of ASD-cancer while suggesting future directions, such as the incorporation of additional data layers and the development of adaptive AI models through continuous learning. Notably, integrating large language models into ASD-cancer could enhance its interpretability, providing more profound insights into oncological research and increasing its influence in cancer subtyping and further analysis.

## INTRODUCTION

Deep learning is transforming cancer data analytics by facilitating the integration of multi-omics data, thereby significantly improving diagnosis, classification, and personalized treatment strategies (1–4). The ASD-cancer framework exemplifies this progress by utilizing autoencoders to extract survival-related features, categorize various cancer subtypes, and identify potential biomarkers (5). It combines gene expression data from The Cancer Genome Atlas (TCGA) with tumor microbiome profiles using Poore et al.’s microbiome profiling technique (6). The framework is validated through external cohort testing with colon cancer data from Qatar and liver cancer data from China (7, 8). However, integrating multi-omics data, particularly tumor microbiome and transcriptome profiles, remains a significant challenge in cancer research (9–12). As discussed in the ASD-cancer study, conventional methods like principal component analysis (PCA) could not effectively capture the complexity of multi-omics data sets (13, 14). This limitation hinders the discovery of intricate biological patterns, highlighting the need for more advanced analytical approaches. ASD-cancer overcomes these limitations, enabling meaningful feature extraction and deeper biological insights. By identifying survival subtypes across 20 cancer types, ASD-cancer enhances risk stratification, informs clinical decisions, and advances personalized oncology, marking a significant step toward precision medicine.

## ASD-CANCER FRAMEWORK: A DEEP LEARNING APPROACH FOR TUMOR ANALYSIS

**Fig 1:**
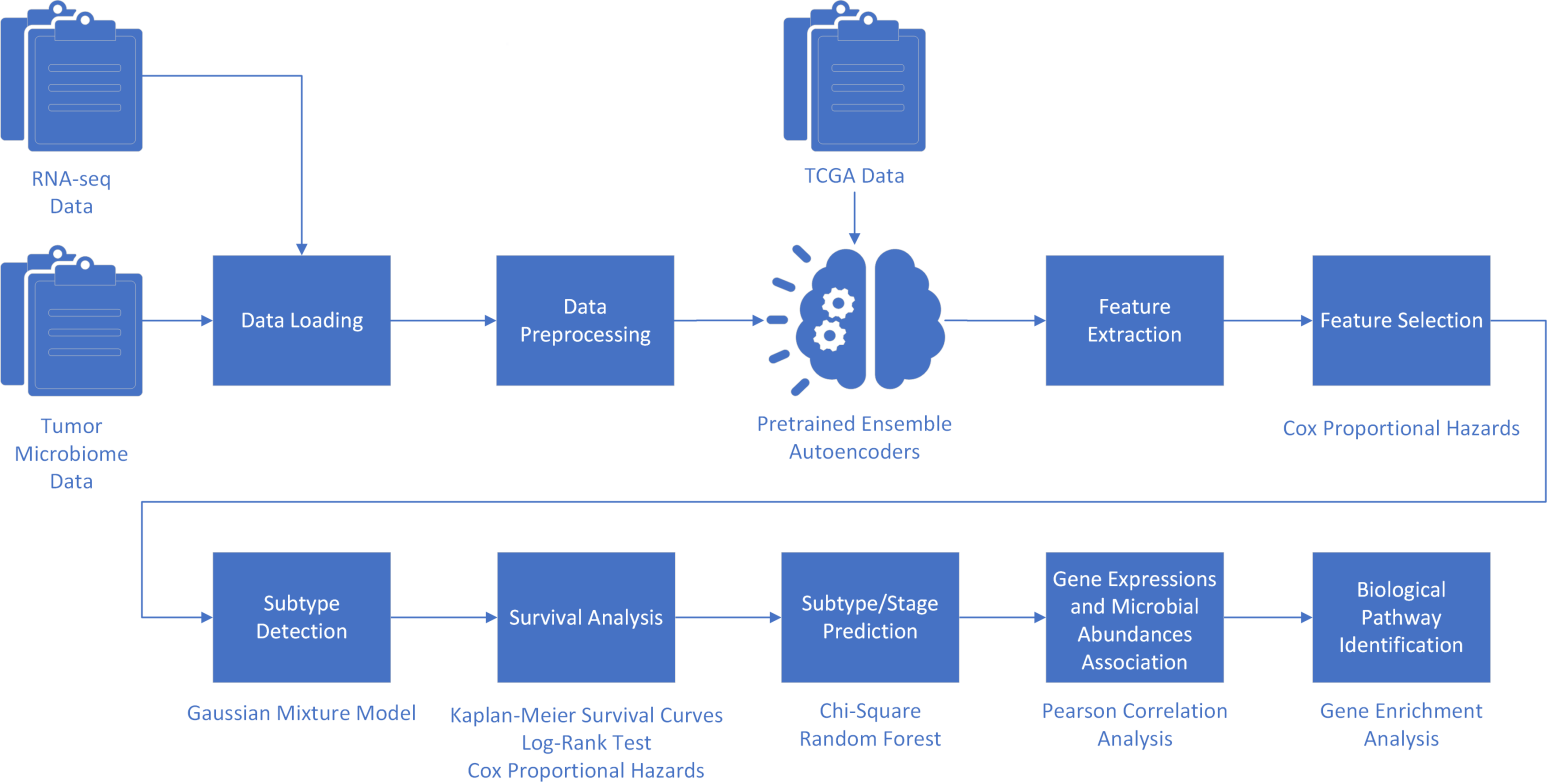
Data flow diagram of ASD-cancer. The ASD-cancer framework integrates multi-omics data, deep learning, and statistical modeling to identify cancer subtypes and assess survival outcomes. The workflow begins with data loading and preprocessing RNA-seq and tumor microbiome data, incorporating TCGA data sets. Pre-trained ensemble autoencoders extract latent features, which undergo feature selection using Cox proportional hazards regression to retain survival-associated biomarkers. Subtype detection is conducted using GMM, followed by survival analysis that includes Kaplan-Meier survival curves, log-rank tests, and Cox regression to validate subtype distinctions. Subtype and stage prediction are conducted using the chi-square test and random forest classifier. Pearson correlation analysis examines associations between gene expression and microbial abundances, while gene enrichment analysis identifies biological pathways and potential biomarkers for cancer prognosis and treatment.

The ASD-cancer framework follows a structured, multiphase data processing pipeline. This method integrates autoencoders with random forest and various bioinformatics techniques, forming a semi-supervised framework for cancer subtype stratification and possible biomarker identification (see Fig. 1). This pipeline can be categorized into the following phases: data input and preprocessing, feature extraction and selection, subtype detection and survival analysis, subtype and clinical stage prediction, gene expression and microbial abundance association, and biological pathway identification.

The pipeline integrates RNA-seq and tumor microbiome data as new or test data. Data preprocessing ensures consistency and quality across multi-omics data sets through normalization.

Subsequently, autoencoders extract features from transcriptomic and microbiome data, capturing nonlinear relationships and reducing noise. Incorporating TCGA data sets enhances feature extraction effectiveness and improves model generalizability for downstream analyses without retraining. The resulting 3,000 latent features per cancer type provide a richer biological context compared to traditional dimensionality reduction techniques like PCA. Cox proportional hazards regression filters out non-informative features, ensuring that only statistically significant survival-associated biomarkers contribute to subtype detection.

Following feature selection, the Gaussian mixture model (GMM) clusters patient data into biologically distinct survival subtypes, optimizing clustering stability through silhouette scoring. Then, Kaplan-Meier survival curves, log-rank tests, and Cox regression perform the prognostic relevance analysis of these subtypes, confirming survival differences between these cancer types.

After survival analysis, the chi-square test and random forest classifier are employed to predict cancer subtypes and clinical stages using the features identified in prior steps. This semi-supervised learning approach integrates the large-scale TCGA data set with smaller, heterogeneous cohorts, enhancing subtype classification generalization by improving feature quality and reducing noise.

Beyond subtype classification, Pearson correlation analysis is applied to identify associations between gene expression profiles and microbial abundances, offering a deeper understanding of potential tumor-microbiome interactions. This step aims to identify molecular signatures associated with cancer progression.

Finally, gene enrichment analysis is performed to identify molecular pathways and functional annotations associated with detected subtypes. This final step reveals potential biomarkers and mechanistic insights that may inform cancer prognosis and therapeutic strategies.

## FUTURE DIRECTIONS FOR DEEP LEARNING IN ONCOLOGY

The ASD-cancer framework has effectively showcased the potential of transfer learning implementation for enhanced multi-omics cancer data analysis. Future research could involve additional data layers, such as proteomics, metabolomics, medical imaging, and clinical data, to further test the effectiveness of transfer learning with even more data modalities. Expanding data integration in this way offers an endless opportunity to deepen our understanding of cancer dynamics, further addressing critical gaps and uncovering new insights.

In addition to data integration, another crucial area to enhance this study is the incorporation of adaptive deep learning frameworks for continuous learning. Building on the foundations of ASD-cancer, these continuous learning modules connect live clinical and multimodal data for real-time predictive analysis in alignment with the latest cancer research and treatment investigations.

Enhancing interpretability is another critical direction for ASD-cancer improvement. While the framework has shown impressive performance in survival subtyping and classification, the intermediate and final outputs require greater explainability. One promising approach is integrating large language models and ASD-cancer to produce natural language explanations of these outputs (15). This integration would help to connect the gap between complex data sets, AI-driven insights, and clinical decision-making, potentially making results more intuitive and translating ASD-cancer’s findings into actionable interventions.

## CONCLUSION

The ASD-cancer framework integrates deep learning, machine learning, and bioinformatics while utilizing TCGA data through transfer learning to investigate multi-omics cancer data, aiming to enhance predictive accuracy and result reliability. Its semi-supervised architecture extracts survival-related features from such complex data sets, identifying distinct subtypes across 20 cancer types. The framework outperforms the baseline model in cancer risk stratification and provides biologically meaningful insights into host gene expression-microbiome interactions. The ASD-cancer framework offers a robust foundation for understanding tumor microenvironment dynamics and enhancing the reliability of risk stratification. By leveraging its artificial neural network architecture to integrate diverse multi-omics data, ASD-cancer serves as a valuable and scalable platform for understanding tumor microenvironment dynamics, improving risk stratification, and guiding future investigations into personalized treatment strategies.
